# Weight Loss Associated With Different Patterns of Self-Monitoring Using the Mobile Phone App My Meal Mate

**DOI:** 10.2196/mhealth.4520

**Published:** 2017-02-02

**Authors:** Michelle C Carter, Victoria J Burley, Janet E Cade

**Affiliations:** ^1^ Nutritional Epidemiology Group School of Food Science and Nutrition University of Leeds Leeds United Kingdom

**Keywords:** self-monitoring, mobile phone, obesity, weight loss

## Abstract

**Background:**

Obesity is a major global public health issue due to its association with a number of serious chronic illnesses and its high economic burden to health care providers. Self-monitoring of diet has been consistently linked to weight loss. However, there is limited evidence about how frequently individuals need to monitor their diet for optimal weight loss.

**Objective:**

The aim of this paper is to describe app usage frequency and pattern in the mobile phone arm of a previously conducted randomized controlled trial. The relationship between frequency and pattern of electronic dietary self-monitoring and weight loss is also investigated.

**Methods:**

A randomized pilot trial comparing three methods of self-monitoring (mobile phone app, paper diary, Web-based) was previously conducted. Trial duration was 6 months. The mobile phone app My Meal Mate features an electronic food diary and encourages users to self-monitor their dietary intake. All food consumption data were automatically uploaded with a time and date stamp. Post hoc regression analysis of app usage patterns was undertaken in the My Meal Mate group (n=43; female: 77%, 33/43; white: 100%, 43/43; age: mean 41, SD 9 years; body mass index: mean 34, SD 4 kg/m^2^) to explore the relationship between frequency and pattern of electronic dietary self-monitoring and weight loss. Baseline characteristics of participants were also investigated to identify any potential predictors of dietary self-monitoring.

**Results:**

Regression analysis showed that those in the highest frequency-of-use category (recorded ≥129 days on the mobile phone app) had a −6.4 kg (95% CI −10.0 to −2.9) lower follow-up weight (adjusted for baseline weight) than those in the lowest frequency-of-use category (recorded ≤42 days; *P*<.001). Long-term intermittent monitoring over 6 months appeared to facilitate greater mean weight loss than other patterns of electronic self-monitoring (ie, monitoring over the short or moderate term and stopping and consistently monitoring over consecutive days). Participant characteristics such as age, baseline weight, sex, ethnicity, conscientiousness, and consideration of future consequences were not statistically associated with extent of self-monitoring.

**Conclusions:**

The results of this post hoc exploratory analysis indicate that duration and frequency of app use is associated with improved weight loss, but further research is required to identify whether there are participant characteristics that would reliably predict those who are most likely to regularly self-monitor their diet.

**ClinicalTrial:**

ClinicalTrials.gov NCT01744535; http://clinicaltrials.gov/ct2/show/NCT01744535 (Archived by WebCite at http://www.webcitation.org/6FEtc3PVB)

## Introduction

Obesity is associated with a range of serious and chronic conditions and is estimated by the World Health Organization to be the fifth leading risk for global deaths [1]. In 2008, 1.4 billion adults across the globe were estimated to be overweight and, of these, over 500 million were obese [1]. In the United Kingdom, the economic cost of obesity is immense with an estimated £4.2 billion annual spend by the National Health Service in 2007 [2]. The behavioral approach to obesity has the underlying assumption that dietary and physical activity behaviors are learned and can be modified by changing the preceding event/trigger for the behavior and by manipulating the consequences [3]. A review of behavioral interventions for weight loss showed that lifestyle interventions resulted in average weight loss equivalent to 11% of initial body weight in the short term [4].

Self-monitoring has been ascribed great importance in behavioral approaches to obesity and has been described as the “centerpiece” [5] and “sine qua non” [3] of weight management strategies. Self-monitoring requires a person to deliberately observe and record their behavior. In the self-regulatory model, self-monitoring focuses attention on behavior by raising the persons’ awareness, offering them the opportunity to adjust behavior as necessary to achieve their goal [6]. Traditionally, studies have investigated dietary self-monitoring using paper diaries [7,8], but as technology improves, researchers have also investigated self-monitoring using handheld electronic devices such as portable microcomputers [9-11], personal digital assistants (PDAs) [12,13], PDAs with Subscriber Identity Module (SIM) cards [14,15], or mobile phones [16].

A systematic review of the self-monitoring and weight loss literature found 22 eligible studies published between 1993 and 2009 [5]. Weight loss was found to be consistently statistically significantly associated with self-monitoring of diet. The review highlighted that there is disparity in the way that adherence to self-monitoring is measured in different studies and many are reliant on assessment by self-report leaving a paucity of information about exactly how much self-monitoring is required for weight loss. There is also a lack of generalizability of findings given that studies predominantly consist of white women. It is questionable as to how effective and acceptable self-monitoring interventions are to a more diverse audience.

It is not yet fully understood whether dietary self-monitoring needs to be conducted over the long term to aid weight loss or whether there is a “learning effect” such that self-monitoring needs only to occur for a short time for permanent changes to be implemented. It is also not known whether dietary self-monitoring can be effective if done intermittently or whether it must be done consecutively on a daily basis for optimum effect. Such information would be useful because it could help to guide individuals on how much dietary self-monitoring they need to do to facilitate their weight loss effort. As technology advances, there is exciting potential for more objective assessment of self-monitoring given that electronic records can be time and date stamped.

One study has provided some valuable data in this area by investigating how a PDA was used for dietary self-monitoring in a weight loss trial. The Self-Monitoring and Recording using Technology (SMART) trial conducted by Burke et al [17] compared weight loss in 210 participants over 2 years [17]. Participants were randomized to one of three dietary self-monitoring arms: a paper diary, PDA, and PDA with feedback. Adherence to dietary self-monitoring was defined as the percentage of days with adequate calories recorded and investigated within three categories (<30%, 30%-59%, ≥60%). The trial found that regardless of group, those who were adherent 60% or more of the time lost more weight than those adherent less than 30% of the time (*P*<.001). However, weight loss at 18 months in the two highest categories of adherence to dietary self-monitoring in all groups (30%-59% and ≥60%) was similar. The researchers suggested that in this case lower levels of adherence to dietary self-monitoring were sufficient to produce the same weight change results as higher levels after this duration of self-monitoring. Because PDAs have now largely been superseded by mobile phones and smartphones, the analysis in this paper will build on the prior evidence by investigating how participants used a mobile phone app for weight loss. The findings presented are a post hoc analysis of the data collected in a pilot trial of My Meal Mate a mobile phone app for weight loss [18]. The aim of this paper is to describe app usage frequency and patterns in the My Meal Mate arm of the My Meal Mate pilot randomized controlled trial. The relationship between frequency and pattern of electronic dietary self-monitoring and weight loss has also been investigated. This work is innovative because the researcher-controlled app provides objective time-stamped data, which allows for a unique exploration of electronic dietary self-monitoring by participants in a 6-month weight loss trial. Therefore, this topic is potentially of interest to the health community and also to the wider quantified self-community.

## Methods

My Meal Mate is an evidence-based mobile phone app designed to facilitate weight loss and has been investigated in a pilot randomized trial [18]. A detailed description of the My Meal Mate intervention has been discussed elsewhere [19] as have the methods and results of the My Meal Mate pilot trial [18]. Briefly, My Meal Mate features an electronic food diary and users are required to select and log food and drink items from a 23,000-item database [18,20]. My Meal Mate was programmed so that all food consumption data were automatically uploaded with a time and date stamp. This presented an opportunity to capture objective information about how people used the electronic diary to self-monitor their diet. A pilot trial was conducted whereby 128 overweight or obese participants were randomized to one of three different methods of dietary self-monitoring; My Meal Mate mobile phone app (participants received a HTC Desire mobile phone with the app predownloaded), paper diary, and online food diary. Because this was a pilot trial, the key outcomes under consideration were feasibility and acceptability; as such, the trial was not statistically powered to detect a particular change in weight. Therefore, a formal sample size calculation was not considered appropriate and the final sample size was a pragmatic decision.

Trial volunteers were recruited by email, intranet, and posters from large local employers. The trial had minimal contact in that participants did not receive any dietary advice and were advised to use the self-monitoring intervention for the first week at least and then as often as they pleased. Participants returned for follow-up at 6 weeks and 6 months. Height, weight, and percentage body fat were measured at three time points (baseline, 6 weeks, and 6 months) by fieldworkers blinded to intervention group. A number of self-administered demographic questionnaires were also completed. A 20-item scale was used to measure conscientiousness. Conscientiousness has been described as “a tendency to be organized, strong-willed, persistent, reliable, and a follower of rules and ethical principles” [21]. The scale was taken from the International Personality Item Pool website, which hosts a freely available inventory of personality measures [22,23]. Participants were requested to self-report how much they agreed with each item (eg, “I pay attention to detail”) on a Likert scale of one to five. Conscientiousness is one of five domains that make up the five-factor model of personality (along with extroversion, agreeableness, neuroticism, and openness) [24]. Conscientiousness, in particular, has been identified as being negatively associated with a number of health-related behaviors, such as tobacco use, diet and physical activity, and drug use [25]. Consideration of future consequences (CFC) was also measured using a 12-item scale that measured “the extent to which people consider the potential distant outcomes of their current behaviors and the extent to which they are influenced by these potential outcomes” [26]. Respondents were asked to rate how characteristic of them a particular behavior was on a Likert scale from one to five. The items were statements such as “I only act to satisfy immediate concerns, figuring the future will take care of itself.”

The analysis discussed in this paper is a post hoc analysis focusing specifically on those participants enrolled in the My Meal Mate arm of the trial (n=43). The relationship between dietary self-monitoring (frequency and pattern) and weight loss was investigated.

### Ethical Approval

This study was conducted according to the guidelines laid down in the Declaration of Helsinki and all procedures involving human subjects/patients were approved by the University of Leeds, Faculty of Medicine and Health Research Ethics Committee (ethics reference number: HSLTLM/10/002). Written informed consent was obtained from all participants.

### Statistical Analysis

Analyses were performed using STATA statistical software version 11 (StataCorp, College Station, TX, USA). Descriptive statistics were used to present baseline characteristics of participants in the My Meal Mate arm of the trial. Throughout the analysis, a complete day of dietary self-monitoring was considered to be one with a biologically plausible energy (kilocalorie [kcal]) intake recorded (≥500 and ≤5000 kcal/≥2093 and ≤20,934 kilojoules).

### Frequency of Dietary Self-Monitoring as a Predictor of Follow-Up Weight at 6 Months

The differences between participants in terms of frequency of dietary self-monitoring were investigated for a number of characteristics measured at baseline. Descriptive statistics are displayed. Statistically significant differences were assessed between the three categories of frequency of monitoring by a one-way ANOVA (where the variable was found to be normally distributed and other assumptions of the test were met) or the nonparametric equivalent Kruskal-Wallis test as appropriate.

The frequency-of-use variable for the My Meal Mate group (number of days using the app for dietary self-monitoring) is a continuous variable; however, its distribution was found to be U-shaped and did not improve after log transformation making it unsuitable to be treated as a continuous variable in a regression analysis. For analysis, the variable was split so that it could be treated as a categorical variable. Due to the distribution of the data, the variable was cut at three points to make categories (low, moderate, and high frequency of use). The variable was cut automatically by STATA at three points, which gave an equal number of participants in each group. This gave a definition of low-frequency use as 42 days or less (n=13), moderate-frequency use as 43 days to 128 days (n=15), and high-frequency use as 129 days or more (n=15) with dietary data recorded (≥500 and ≤5000 kcal). An intention-to-treat regression analysis that used weight at follow-up (with baseline observation carried forward for any missing data) as the outcome variable and the frequency-of-use category as a predictor variable was conducted. The model was adjusted for baseline weight, but no other adjustments were made given that no variables were found to differ in a statistically significant way between the categories.

### Pattern of Dietary Self-Monitoring as a Predictor of Follow-Up Weight at 6 Months

The frequency of My Meal Mate use is an overall count of days with dietary self-monitoring over the course of the trial, but it misses information about the distribution of the days. For example, persons A and B may have both recorded 50 days on the My Meal Mate app, but person A may have monitored consecutively at the beginning of the trial for 50 days and then stopped, whereas person B may have recorded 50 days intermittently over the course of the 6-month period. Therefore, pattern of monitoring in relation to weight loss was investigated. The distribution of data collected on My Meal Mate was visually inspected and used to divide the participants into discrete patterns of self-monitoring. Differences between the patterns of dietary self-monitoring in a number of key variables measured at baseline were investigated using appropriate inferential statistics (one-way ANOVA or Kruskal-Wallis test as appropriate) and a regression analysis conducted with pattern of adherence as a categorical predictor of follow-up weight (adjusted for baseline weight).

## Results

### Baseline Characteristics of Participants Enrolled in the My Meal Mate Pilot Trial

Table 1 shows the baseline characteristics of all participants enrolled in the My Meal Mate pilot trial. This paper focuses on participants in the My Meal Mate group, but all three groups in the trial are shown for comparison. Table 1 also shows the main outcomes from the My Meal Mate pilot trial. The results of the trial are reported elsewhere [18], but have been included here to compare My Meal Mate to the other arms in the trial. Of the 43 adults in the My Meal Mate group, more than three-quarters (33/43) were female and all (43/43) were white. The mean age of the My Meal Mate group participants was 41 (SD 9) years and more than half (32/43) were employed in managerial and professional occupations. The mean participant body mass index (BMI) in the My Meal Mate group was 34 (SD 4) kg/m^2^.

**Table 1:** Baseline characteristics of all participants enrolled in the My Meal Mate pilot trial.

Participant characteristics	Mobile phone (n=43)	Diary (n=43)	Website (n=42)
Age (years), mean (SD) [95% CI]	41.2 (8.5) [38.6-43.9]	42.5 (8.3) [39.9-45.0]	41.9 (10.6) [38.6-45.2]
Weight (kg), mean (SD) [95% CI]	96.4 (16.0) [91.9-101.8]	97.9 (18.7) [92.2-103.6]	96.4 (19.9) [90.2-102.6]
Body mass index (kg/m^2^), mean (SD) [95% CI]	33.7 (4.2) [32.4-35.0]	34.5 (5.7) [32.7-36.2]	34.5 (5.6) [32.7-36.2]
Sex (female), n (%)	33 (77)	33 (77)	33 (79)
Race (white), n (%)	43 (100)	35 (83)	39 (93)
Smoking status (current smokers), n (%)	2 (5)	8 (19)	2 (5)
Occupation (managerial professions),^a^ n (%)	32 (74)	22 (51)	20 (49)
Has a university degree, n (%)	31 (72)	24 (56)	22 (53)
Owns a mobile phone, n (%)	18 (42)	19 (44)	14 (34)

^a^The occupation variable was dichotomized; it was originally measured as (1) managerial and professional occupations, (2) intermediate occupations, (3) small employers and own account workers, (4) lower supervisory and technical occupation, and (5) semiroutine and routine occupations.

### Use of the My Meal Mate App and Total Weight Change

Over the 6-month trial period, participants used the My Meal Mate app for a median 82 (IQR 28-172) days to record their intake. In all, 40 of 43 participants returned to be weighed at 6 months. All participants completed at least one day of dietary self-monitoring and only two participants completed less than 7 days of dietary self-monitoring. Within the My Meal Mate group, using an intention-to-treat analysis (with baseline observation carried forward for the three missing follow-up weights), the mean weight change at 6 months was −4.6 kg (95% CI −6.2 to −3.0). For trial completers only (n=40), the mean weight change was −5.0 kg (95% CI −6.7 to −3.3).

### High-, Moderate-, and Low-Frequency Users of My Meal Mate

Table 2 presents differences in key variables measured at baseline between the different categories of frequency of My Meal Mate use. There were no statistically significant differences found between the frequency-of-use categories for any of these key variables. There was a suggestion of a trend for greater weight loss at 6 weeks with self-monitoring, but this was not statistically significant. Table 3 presents the results of a regression analysis investigating frequency-of-use category as a predictor of follow-up weight at 6 months (adjusted for baseline weight). Those in the highest adherence category (recorded ≥129 days on the My Meal Mate app) had a −6.4 kg (95% CI −10.0 to −2.9) lower follow-up weight (adjusted for baseline weight) than those in the lowest adherence category (recorded ≤42 days). This difference was found to be statistically significant (*P*=.001). The difference in follow-up weight was not found to be statistically significantly different between those in the moderate category of adherence and those in the low category of adherence (−1.8 kg, 95% CI −5.3 to 1.8, *P*=.33). If the dummy variable was recoded so that the medium adherence category was the reference category (43-128 days), the high adherence category was found to have a −4.7 kg (95% CI −8.2 to −1.1) lower follow-up weight (adjusted for baseline weight) (*P*=.01). However, it is worth noting that the confidence intervals are fairly wide because the sample is very small.

**Table 2:** Baseline characteristics of different categories of subsequent My Meal Mate use.

Participant characteristic	Category of frequency of My Meal Mate use (days of dietary self-monitoring)		
	Low (≤42) n=14	Moderate (≥43 to ≤128) n=14	High (≥129) n=14
Age (years),^a^ mean (95% CI)	39.1 (34.4, 43.8)	40.4 (34.9, 45.8)	44.1 (39.7, 48.4)
Baseline weight (kg),^a^ mean (95% CI)	96.8 (88.2, 105.3)	100.5 (90.1, 110.9)	93.5 (84.8, 102.1)
Baseline BMI (kg/m^2^),^a^ mean (95% CI)	33.8 (31.1, 36.4)	34.8 (32.3, 37.2)	32.7 (30.6, 34.9)
Conscientiousness score,^a^ mean (95% CI)	76.5 (66.1, 84.8)	76.1 (70.0, 82.2)	81.2 (76.0, 86.5)
Score for CFC,^a,b^ mean (95% CI)	31.9 (28.2, 35.6)	33.7 (29.4, 38.0)	28.2 (23.8, 32.5)
Weight change 6 weeks,^a^ mean (95% CI)	−1.8 (−2.7, −0.8)	−3.4 (−5.0, −1.7)	−3.6 (−5.0, −2.2)
Sex (female),^c^ n (%)	11 (79)	10 (71)	12 (80)
Race (white),^c^ n (%)	14 (100)	14 (100)	15 (100)
Managerial and professional occupation,^c^ n (%)	10 (71)	10 (71)	12 (80)
Has a university degree,^c^ n (%)	12 (86)	8 (57)	11 (73)

^a^Significant differences between the three categories of adherence assessed by one-way ANOVA.

^b^CFC: consideration of future consequences.

^c^Significant differences assessed by Kruskal-Wallis.

**Table 3:** Regression analysis of category of My Meal Mate use as a predictor of follow-up weight (adjusted for baseline weight) in the My Meal Mate arm of the pilot trial.

Category of adherence	n	Weight loss coefficient (kg) (95% CI)	*P* value
Low (≤42 days of dietary self-monitoring)	14	Reference	—
Moderate (≥43 days to ≤128 days of dietary self-monitoring )	14	−1.8 (−5.3, 1.8)	.33
High (≥129 days of dietary self-monitoring)	15	−6.4 (−10.0, −2.9)	.001

### Pattern of My Meal Mate Use Over the Course of the Trial

**Figure 1:**
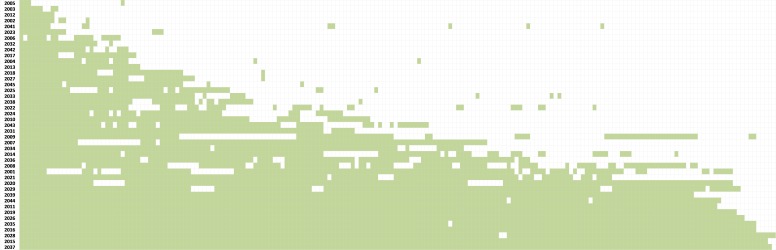
Distribution of days of dietary recording on My Meal Mate for each participant (n=43) over the course of the 6-month My Meal Mate pilot trial. The x-axis is the My Meal Mate ID number that was automatically assigned to the participant and the y-axis is dietary self-monitoring in days. Each green shaded box is a day with ≥500 and ≤5000 kcal energy recorded on My Meal Mate.

Figure 1 shows the distribution of daily dietary recording by each individual over the course of the trial. The distribution of data in Figure 1 has been visually inspected and used to divide the participants into four discrete patterns of self-monitoring. This categorization is based on the following limits, which were true as a result of the observation of Figure 1:

1. Stopped early: last diary entry before 31 days;

2. Moderate-term monitoring: last entry before 92 days (approximately 3 months);

3. Long-term intermittent monitoring: monitored over the long term (3-6 months), but intermittently with breaks (a break is at least 1 day); and

4. Long-term consecutive monitoring: monitored mostly consecutively over the long term (no more than four breaks and breaks never longer than 10 days).

The differences between the patterns of dietary self-monitoring in a number of key variables at baseline are displayed in Table 4. No statistically significant differences were found between the four different patterns of monitoring on a number of variables except for weight change at 6 weeks. A regression analysis was conducted using follow-up weight as the outcome variable and pattern of adherence category as a predictor (adjusting for baseline weight). The regression output can be seen in Table 5.

The results of the regression analysis show that those who monitored intermittently over the long term had a −7.5 kg (95% CI −11.6 to −3.4) lower follow-up weight (adjusted for baseline weight) than those who stopped monitoring completely before 31 days (*P*=.001). The difference in follow-up weight was not found to be statistically significantly different between the other two categories compared to the “early stoppers” reference group. The confidence intervals around the coefficients were wide, which is likely reflective of the small sample size within categories once the variable was split.

**Table 4:** Investigation of the differences between patterns of My Meal Mate use for key variables measured at baseline in the My Meal Mate group.

Participant characteristic	Pattern of My Meal Mate use^a^			
	Stopped early (n=11)	Stopped before 92 days (n=9)	Intermittent over long term (n=11)	Consecutive over long term (n=12)
Age (years),^b^ mean (95% CI)	36.7 (31.6, 41.9)	42.2 (34.2, 50.3)	42.8 (37.7, 48.0)	43.2 (38.1, 48.3)
Baseline weight (kg),^b^ mean (95% CI)	96.4 (84.8, 107.9)	94.5 (80.4, 108.6)	96.3 (85.2, 107.5)	99.5 (90.4, 108.6)
Baseline BMI (kg/m^2^),^b^ mean (95% CI)	33.9 (30.9, 37.0)	31.2 (29.9, 32.6)	33.6 (30.5, 36.7)	35.5 (32.7, 38.2)
Conscientiousness score,^b^ mean (95% CI)	75.6 (64.4, 86.8)	78.0 (67.9, 88.1)	79.6 (72.9, 86.4)	78.2 (73.2, 83.2)
Score for CFC,^b,c^ mean (95% CI)	33.0 (30.4, 35.6)	30.6 (24.3, 36.9)	29.1 (23.6, 34.6)	32.5 (26.9, 38.2)
Weight change 6 weeks^b^ mean (95% CI)	−1.4 (−2.9, −0.1)	−2.6 (−3.9, −1.4)	−4.2 (−6.5, −1.9)	−3.4 (−4.4, −2.4)
Sex (female),^d^ n (%)	9 (81)	5 (56)	9 (81)	10 (83)
Race (white),^d^ n (%)	11 (100)	9 (100)	11 (100)	12 (100)
Managerial and professional occupation,^d^ n (%)	8 (73)	9 (100)	10 (91)	5 (42)
Has a university degree,^d^ n (%)	7 (64)	7 (78)	9 (82)	8 (67)

^a^Stopped early: recorded <31 days; consecutive over long term: monitored over the 6-month period with no more than four breaks of no more than 10 days at a time.

^b^Significant differences between the four patterns of use assessed by one-way ANOVA.

^c^CFC: consideration of future consequences.

^d^Significant differences assessed by Kruskal-Wallis.

**Table 5:** Regression analysis of pattern of My Meal Mate use as a predictor of follow-up weight (adjusted for baseline weight) in the My Meal Mate arm of the pilot trial.

Pattern of adherence	n	Weight loss coefficient (kg) (95% CI)	*P* value
Stopped early	11	Reference	—
Moderate	9	–3.1 (−7.4, 1.2)	.16
Long but intermittent	11	–7.5 (−11.6, −3.4)	.001
Long consecutive	12	–3.2 (−7.2, 0.8)	.12

## Discussion

A post hoc analysis investigating the relationship between dietary self-monitoring (using a mobile phone app) and weight loss has been presented. High-frequency users of the My Meal Mate app (recorded ≥129 days of intake) were found to have a −4.7 kg (95% CI −8.2 to −1.1, *P*=.001) lower mean follow-up weight (adjusted for baseline) than moderate users (43-128 days) and a −6.4 kg (95% CI −10.0 to −2.9, *P*=.001) lower mean follow-up weight than low-frequency users (≤42 days). The difference in follow-up weight between moderate- and low-frequency users was not found to be statistically significant (*P*=.33). Those who monitored intermittently over the whole course of the trial had a −7.5 kg (95% CI −11.6 to −3.4, *P*=.001) lower mean follow-up weight (adjusted for baseline) than those who monitored for a short time and stopped early. Those who monitored for a moderate time and then stopped and those who monitored consecutively over the 6 months did not have a statistically significantly greater mean weight loss than those who monitored for a short time and stopped (*P*=.16 and *P*=.12, respectively). These results provide preliminary evidence that continuous self-monitoring may not be necessary for weight loss.

Participant characteristics such as age, sex, conscientiousness, and CFC were not found to predict extent of self-monitoring as reflected by number of days of app use. However, our sample size may have been too small to detect differences. A post hoc power calculation shows that based on the follow-up values from the mobile phone and paper diary group in the My Meal Mate pilot trial, the sample size had 90% power to detect a statistically significant difference of 13 kg in follow-up weight between two groups and 80% power to detect a difference of 11 kg (at the 5% significance level). The sample size in the trial (n=43 in each arm) had 10% power to detect the actual difference in follow-up weight found between groups.

### The Relationship Between Frequency of Dietary Self-Monitoring and Weight Loss

Dietary self-monitoring is an important outcome because it has been consistently linked to weight loss [5,8,27]. The frequency-of-use findings presented in this paper are supportive of the findings of the SMART trial which analyzed different categories of adherence to a PDA, PDA with feedback, and a paper diary [13,17]. The SMART trial reported that weight loss was greater (across groups) for those in the highest categories of adherence to dietary self-monitoring (≥60% adherent) than those in the lowest categories (≤30% adherent). For example, in the PDA and feedback group (n=70), mean percentage weight change at 18 months was −10% (SD 9%) in the highest adherence category (≥60% adherent), −12% (SD 9%) in the medium adherence category (30%-59% adherent), and −3% (SD 7%) in the low adherence category (<30%). Burke et al [17] found that moderate- and high-frequency users had lost roughly equivalent amounts of weight at 18 months, whereas the findings from this trial of My Meal Mate suggest that high-frequency users had lost a statistically significantly greater amount of weight than moderate users. At 6 months in the SMART trial, it appears that the difference in weight loss between high- and moderate-frequency users was wider. For example, in the PDA plus feedback arm, those in the high adherence category (≥60% adherent) had a −9% (SD 7%) mean weight change compared to a −2% (SD 5%) mean weight change in the medium adherence category (30%-59% adherent). Because the trial of My Meal Mate was only for 6 months, it is not known whether the weight loss seen would continue to be maintained in the long term. Therefore, the findings from the SMART trial are interesting because the optimum amount of electronic dietary self-monitoring for weight loss in the short term may be different from the optimum amount for long-term weight maintenance.

### The Relationship Between Pattern of Dietary Self-Monitoring and Weight Loss

A unique aspect of the analysis presented here is that the pattern of dietary self-monitoring was considered in addition to the frequency. This exploratory analysis does suggest that long-term intermittent monitoring was associated with a greater weight loss and that monitoring in the short or moderate term and stopping completely was not enough to imbue the user with the necessary changes to lose weight by 6 months. Surprisingly perhaps, long-term intermittent monitoring was more effective for weight loss than those who used the My Meal Mate app fastidiously with consecutive days of monitoring and few breaks. However, the small numbers within categories do give wide confidence intervals so results must be interpreted with caution.

There is a gap in knowledge about the optimum frequency and pattern of self-monitoring necessary for successful weight loss. These findings suggest that there may be some kind of “learning effect” in the intermittent group that they did not need to use the My Meal Mate app to track calories every single day, but were perhaps self-managing the days when they needed extra help to track over the long term. It could be speculated that the group of individuals who monitored consecutively every day relied on the phone to self-monitor, but were not learning as much about their intake or feeling as confident about having days of nontracking when they were responsible for their own instinctive self-management. Perhaps those that monitored intermittently over the 6 months were still sufficiently invested in the process of self-monitoring to carry it out over the long term, but during this time their awareness of their dietary intake and self-sufficiency had increased so that they could identify when they needed some more support and could use the diet tracking as and when they needed it. At this stage, this interpretation is conjecture and in a definitive trial with larger numbers, an attempt to classify pattern of self-monitoring in this way would be useful to further investigate how much dietary self-monitoring is necessary.

### Predictors of Dietary Self-Monitoring

There is little evidence to suggest which individual characteristics may or may not be predictive of successful dietary self-monitoring. A range of baseline characteristics were investigated between categories of frequency of dietary self-monitoring and pattern of self-monitoring (including personality traits such as conscientiousness and CFC), but none were found to be statistically significantly different. It may be the case that these factors are genuinely not predictors of dietary self-monitoring or it could be that the sample size was too small to detect such differences. It would be interesting to examine potential predictors of successful dietary self-monitoring in a larger trial. If such characteristics were identified as predictors of successful dietary self-monitoring, it may indicate scope to target those most likely to find it useful.

### The Need for a Consistent Dietary Self-Monitoring Adherence Outcome

Researchers have taken different approaches to measuring frequency of electronic dietary self-monitoring, so direct comparison of results is difficult. For example, frequency of use or adherence to dietary self-monitoring has been measured in the following way by different studies: total number of days with over 900 kcals recorded using a handheld microcomputer [28], number of weekly submissions of PDA records [12], as a binary variable with adherent behavior categorized as more than 50% of weekly calorie goal met [29], percentage of days with plausible intakes recorded on a PDA, sampled for the first and last week of the study [30], and self−reported number of days per week with dietary self-monitoring using a mobile phone app in addition to a podcast and Twitter intervention [31].

Frequency of dietary self-monitoring has been measured differently in each study; therefore, it is difficult to describe a range of adherence across the studies. In this trial, the number of days with a plausible energy intake was intended to be analyzed as a continuous variable. However, given the U-shaped distribution of the variable it was more appropriate to split it into categories rather than treat it as continuous in a regression analysis. Although the frequency-of-use variable is useful for exploratory analysis, it is still a rather crude measure of adherence to dietary self-monitoring because it does not provide details about weekly patterns of monitoring over time. Adherence has been measured differently by other researchers. Burke et al [29] created a binary variable of adherent or nonadherent, which was based on the person recording ≥50% of their weekly calorie goal. This gives a week-by-week pattern of adherence over time. However, this is still quite an arbitrary cut-off given the paucity of evidence about what constitutes successful dietary self-monitoring. The differing approaches to measuring adherence to dietary self-monitoring make comparison between studies difficult and a standard approach is warranted.

### Limitations

Generalizability of the results is limited given that the sample is exclusively of white ethnic origin, predominantly female and mostly employed in managerial/professional occupations. My Meal Mate was a prototype app and participants reported that they frequently encountered bugs that caused the app to close. This may have affected participant engagement. As a pilot, the trial was not statistically powered to detect a particular change in weight and the primary outcomes were feasibility and acceptability measures. Therefore, the results from this post hoc analysis need to be interpreted with caution given the small numbers in the My Meal Mate arm and the multiple testings, which increases the risk of a type 1 statistical error. In addition to the variables measured at baseline, it is acknowledged that there are other potential predictors of dietary self-monitoring which might be investigated when examining frequency of use of a mobile phone app, such as usability, technology acceptance, satisfaction, and ease of use. Usability in particular might be particularly interesting to examine, given that a recent pilot trial found perception of usability to be associated with high adherence to a technology-supported intervention to improve fitness in older adults [32]. Despite these limitations, the results are interesting and are intended to be interpreted as exploratory and hypothesis generating.

### Strengths

The automated time- and date-stamped information collected by the My Meal Mate app is a strength because it allows for objective analysis of dietary self-monitoring. The work presented is also unique in considering not only the frequency of self-monitoring, but also the distribution of monitoring days/pattern of monitoring over time. If more is known about how much self-monitoring is effective and whether monitoring needs to be consecutive or whether breaks in monitoring are acceptable, participants in weight loss trials could be given more prescriptive advice about how best to track their diet and be supported in adherence to self-monitoring.

### Conclusion

A post hoc analysis of the relationship between dietary self-monitoring (frequency and pattern) and weight loss in participants using a mobile phone app to facilitate weight loss has been presented. In this trial, the optimum use of the My Meal Mate app for weight loss appeared to be 129 days or more and intermittently over the long term (3-6 months). Given the small sample size within the My Meal Mate arm of the trial and the dangers of multiple testing, the results from this analysis, although interesting, must be treated with caution.

The investigations conducted in this paper are important because although dietary self-monitoring is associated with weight loss, there is a paucity of information about what to recommend to overweight/obese individuals about the optimal level of monitoring. Electronic means of dietary self-monitoring, such as online dietary assessment systems, PDAs, and mobile phone apps, provide a unique opportunity to investigate self-monitoring behavior objectively. Future research should continue to seek to establish the “optimum dose” for effective dietary self-monitoring and whether certain personality traits are associated with effectiveness of self-monitoring for weight loss.

## Abbreviations

BMI: body mass index
CFC: consideration of future consequences
PDA: personal digital assistant
SMART: Self-Monitoring and Recording using Technology
